# Interaction studies of Alzheimer's Cathepsin B protein with inhibitors in presence and absence of water

**DOI:** 10.1186/1758-2946-6-S1-P23

**Published:** 2014-03-11

**Authors:** Nitin Chitranshi, Pushpendra K Tripathi, Prahlad K Seth

**Affiliations:** 1 Gautam Buddh Technical University, Lucknow 227202, Uttar Pradesh, India; 2 Bioinformatics Centre, Biotech Park, Sector-G, Jankipuram, Lucknow 226021, Uttar Pradesh, India

## Background

The accuracy of ligand-protein docking may be affected by the presence of water molecules on the surface of proteins. Water can form complex bridging networks and can play a critical role in dictating the binding mode of ligands. A recent analysis of high-resolution crystal structures of ligand-protein complexes revealed that 85% of the complexes had one or more water molecules bridging the interaction between ligand and protein. For predicting the binding modes and energies of protein-ligand interactions, molecular docking methods are commonly used. In order to obtain an accurate complex geometry and binding energy estimation, an appropriate method for calculating partial charges is essential. AutoDockTools software, widely used as interface for preparing input files, utilizes the either Gasteiger or Kollman partial charge calculation method for both protein and ligand charge calculations. However, it has already been reported that more accurate partial charge calculation and as a consequence, more accurate docking can be achieved by using quantum chemical methods. In common practice so far, the quantum chemical partial charges were applied to the ligands for docking calculations. The newly developed Mozyme function of MOPAC2009 allows fast partial charge calculation of proteins by quantum mechanical semi-empirical methods. Thus, in the current study, we use the semi-empirical quantum-mechanical partial charge calculations to investigate the interaction energies and polarization effects of the various components of the binding pocket on a set of Cathepsin B protein.

## Results

The docking accuracy was computed by using the original AutoDock scoring function with the set of 19 protein ligand complexes using Gasteiger, AM1 and PM3 partial charge calculation methods. This helped us to compare the effect of the partial charge calculation method on docking accuracy. It was seen that the docking accuracy in regard to complex geometry significantly increased when partial charges of the ligands and proteins were calculated with the semi-empirical PM3 method. Our results demonstrate that (i) the energetic of the key water molecule are more favorable for the binding site in the Cathepsin B protein (ii) Water bridging and triangle formation were seen between the key amino acid residue and the ligand (iii) The internal energy is significant factor for the binding modes of various ligands. It was also observed a statistically significant overall increase in accuracy when water molecules are included during docking simulations. Out of the 19 complexes analyzed in the course of our study, the geometry of 17 complexes were accurately calculated using PM3 partial charges, while the use of Gasteiger charges resulted in only 8 accurate geometries.

**Figure 1:**
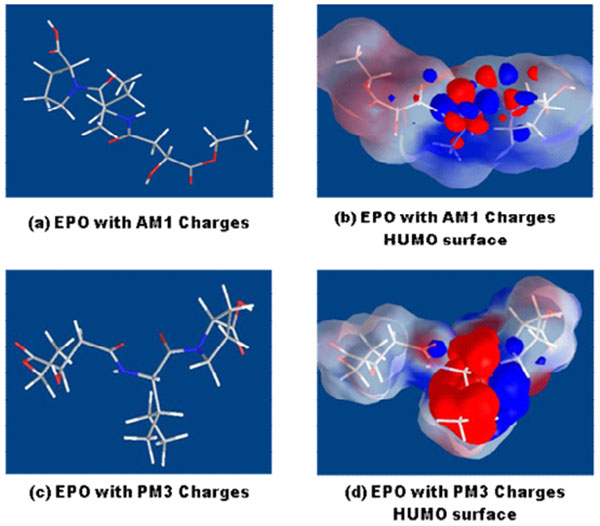
Charge and force field applied to inhibitors for better docking score.

## Conclusion

Our findings indicate that the inclusion of water molecules in ligand-protein docking results in significant increases in docking accuracy when the use of quantum chemical partial charge assignment on both ligand and proteins for predicting the docking simulations.

